# Letter to the Editor: Virtual reality in the treatment of eating and weight disorders

**DOI:** 10.1017/S0033291717001441

**Published:** 2017-10

**Authors:** G. Riva

**Affiliations:** 1 Centro Studi e Ricerche di Psicologia della Comunicazione, Università Cattolica del Sacro Cuore, Milano, Italia; 2 Applied Technology for Neuro-Psychology Lab, Istituto Auxologico Italiano, Milano, Italia

I read with interest the recent paper by Freeman *et al.* exploring the potential of virtual reality (VR) in treating mental health issues (Freeman *et al.* 2017). I agree with the authors that ‘VR has the potential to transform the assessment, understanding, and treatment of mental health problems.’ (p. 1). However, I don't think the overall picture presented by the paper adequately reflects the level of evidence offered by the extant research in the field.

It is well known that the best evidence in healthcare is obtained by the outcomes of randomized controlled trials (RCTs), systematic reviews, and meta-analyses. In September 2016, a paper was published that assessed the 27 available reviews and meta-analyses exploring the efficacy of VR in behavioral health (Riva *et al.* 2016). In that paper, the authors’ findings supported the use of this technology for the treatment of anxiety disorders, stress-related disorders, pain management, and eating and weight disorders. This latter area, i.e. eating and weight disorders, is where the two papers provide different perspectives.

Freeman *et al*. (2017) stated: ‘It has been recognized that the field has very few methodologically strong studies.’ This was true in the past, but the situation is quite different now: three different RCTs (Cesa *et al.* 2013; Marco *et al.* 2013; Manzoni *et al.* 2016) have shown at 1-year follow-up that VR had a higher efficacy than the gold standard in the field, i.e. cognitive–behavioral therapy (CBT).

In fact, the clinical use of VR with these disturbances is based on theory-driven psychological treatment techniques (Ferrer-Garcia & Gutierrez-Maldonado, 2012; Ferrer-Garcia *et al.* 2013; Koskina *et al.* 2013). First, VR can reduce eating-related anxiety during and after exposure to virtual food, helping to disrupt the reconsolidation of adverse, food-related memories (Koskina *et al.* 2013; Pla-Sanjuanelo *et al.* 2015). Second, a recent neuroscientific model of body image disturbances – the Allocentric Lock Theory – suggested that eating disorders may be associated with impairment in the ability to update a stored, negative allocentric (offline) representation of one's body with real-time (online/egocentric), perception-driven inputs (Riva & Gaudio, 2012; Riva, 2014; Dakanalis *et al.* 2016). As demonstrated by two of the above RCTs (Cesa *et al.* 2013; Manzoni *et al.* 2016), the addition of VR sensory training to unlock the body memory (body image rescripting protocol) by increasing the contribution of new, egocentric/internal, somatosensory information directly related to the existing allocentric memory improved the efficacy of CBT at 1-year follow-up.

In their review, Freeman *et al.* (2017) also suggested the use of the body-ownership illusion to enhance the effectiveness of the treatments for eating disorders. Even if I and other researchers (Dakanalis *et al.* 2017; Serino and Dakanalis, 2016) agree that this approach has clinical potential, it is also true that bodily illusions have yet to be tested in an RCT against an active treatment. In addition, the real problem associated with the use of existing bodily illusions (i.e. illusions based on visuo-tactile synchrony) in mental health is the lack of long-term effects. As reported by Freeman *et al.* (2017), the longest follow-up of subjects with eating disorders was just 2 h (Keizer *et al.* 2016), but, in general, the effects of bodily illusions on higher cognitive processes are temporary, even with normal subjects. A possible reason the follow-up time of such subjects has been so short is that most of the existing research on bodily illusions has explored how external information from the body (e.g. vision and touch) is processed and integrated to develop our sense of self. Notwithstanding the success of such advances (Maister *et al.* 2015), we do not perceive our body only through external senses; we also have internal access to it through inner signals, i.e. interoceptive, proprioceptive, and vestibular signals (Blanke, 2012). Recent research has suggested that there is a clear link between mental health disorders and interoceptive/proprioceptive impairments (Tsakiris & Critchley, 2016). In this view, future bodily illusions should bridge VR with bio/neuro-feedback and/or brain/body stimulation technologies to modulate the inner experience of the patient, too (Riva *et al.* 2017). A possible path is ‘Sonoception,’ (Riva *et al*. 2017) which is the use of sound and vibration synchronously with VR for the stimulation of mechanoreceptors in the chest (heart) and in the abdomen (stomach). In conclusion, VR already is a reality in mental health, but more can be done to transform the Latin dictum, ‘*Mens Sana in Corpore Virtuale Sano*’ (a healthy mind in a healthy virtual body), into reality.
